# Direct Healthcare Costs of Moderate and Severe Work-Related Injuries: Estimates from the National Trauma Center of Qatar

**DOI:** 10.3390/ijerph19031609

**Published:** 2022-01-30

**Authors:** Rafael J. Consunji, Ahammed Mekkodathil, Ayman El-Menyar, Amber Mehmood, Brijesh Sathian, Adnan A. Hyder, Nazia Hirani, Aisha Abeid, Hassan Al-Thani, Ruben Peralta

**Affiliations:** 1 Hamad Injury Prevention Program, Hamad Trauma Centre, Department of Surgery, Hamad General Hospital, Hamad Medical Corporation, Doha P.O. Box 3050, Qatar; mekkodathil@yahoo.co.uk (A.M.); aelmenyar@hamad.qa (A.E.-M.); nazia.gadhia@gmail.com (N.H.); AAbeid@hamad.qa (A.A.); Halthani@hamad.qa (H.A.-T.); rperaltamd@gmail.com (R.P.); 2 College of Public Health, University of South Florida, Tampa, FL 33612, USA; amehmood@usf.edu; 3 Geriatrics and Long-Term Care Department, Rumailah Hospital, Hamad Medical Corporation, Doha P.O. Box 3050, Qatar; bsathian@hamad.qa; 4 Milken Institute School of Public Health, The George Washington University, Washington, DC 20052, USA; hydera1@gwu.edu; 5 Department of Surgery, Universidad Nacional Pedro Henriquez Urena, Santo Domingo 1423, Dominican Republic

**Keywords:** work-related injuries, healthcare cost, trauma

## Abstract

Work-related injuries (WRIs) are recognized as a leading cause of admission to the national trauma center of Qatar. A retrospective analysis of trauma registry data and electronic medical records was conducted on a cohort of all WRI patients who were admitted to the Hamad Trauma Center (HTC), in Doha, Qatar, between 2011 and 2017. A total of 3757 WRI patients were treated at the HTC over the 7-year study period. The overall cost for treatment was 124,671,431 USD (18 million USD per year), with a median cost of 19,071 USD. There was a strong positive correlation between the overall cost and hospital-stay cost (r^2^ = 0.949, *p* = 0.00001) and between the overall cost and procedure cost (r^2^ = 0.852, *p* = 0.00001). Motor vehicle crash (MVC) victims who wore seatbelts had significantly lower injury severity, hospital stay and median total costs. A comparison of patients by quartiles of the costs incurred showed that the proportions of MVC victims, pedestrian injuries and mortality were significantly higher in the fourth quartile when compared to other quartiles (*p* < 0.05). These findings suggest that investments in the primary prevention of work-related injuries from falls and MVCs, through proven interventions, should be priorities for occupational safety and health in Qatar.

## 1. Introduction

Work-related injuries (WRIs) remain a significant public health problem, as nearly half of the population across the world (3.5 billion) are workers and hazardous working conditions can increase their risk for injuries [1]. Annually, more than 2 million people die from work-related diseases and injuries. It was also estimated that work-related health issues result in an economic loss of 4–6% of GDP [1]. WRIs are most reported among migrant workers, especially in those from low- and middle-income countries working in the Middle East, Europe and North America, as they are more likely to be employed in high-risk industries [2,3,4,5,6,7,8,9,10]. Qatar is a “receiving” country for migrant workers, with its well-documented human resource needs driven largely by the infrastructure requirements for hosting the 2022 FIFA World Cup. This has put its occupational safety environment under scrutiny from global media despite scientific studies that report dramatic reductions in the incidence of WRIs [11].

An initial analysis of WRIs in Qatar, from 2010 to 2012, revealed that they comprised 29% of all severe injury admissions to the national trauma center, and over half were due to falls; falling objects and motor vehicle crashes (MVCs) were the other leading mechanisms of injury. Only two-thirds of victims used protective devices, and over half were classified as simple laborers [12]. An expanded analysis of WRIs in Qatar further described the difference in patterns between fatal and non-fatal WRIs, with one in four sustaining severe injuries (ISS ≥ 16), and recommended future targeted risk factor analysis and occupational safety interventions [13]. The most extensive analysis of temporal trends in moderate-to-severe work-related injuries in Qatar described a 37% reduction of the incidence of injury per 100,000 workers from 2008 to 2016. The proportion of falls from height decreased during the study period and that from RTIs increased [11].

Cost-of-illness analysis is a way of measuring medical and other costs resulting from a specific disease or condition; this illness analysis may include direct costs, productivity losses and intangible costs of a disease or injury. The latter two costs are generally more complicated to measure than direct costs [14]. Healthcare expenditure in Qatar is considered to be among the highest in the Middle East. It is reported that 2.6 billion was invested in healthcare in 2010; this increased to 4.7 billion USD in 2014 [15,16]. A comprehensive cost analysis of WRIs in Qatar has not been conducted to date. However, Tuma et al. published the landmark study that estimated that the mean cost of care per admitted patient with construction-related falls in Qatar was 16,000 USD in 2008 [17].

This study aimed to describe and analyze the direct cost of inpatient care of moderate-to-severe WRIs admitted to the national trauma center of Qatar, the Hamad Trauma Center (HTC). Specific objectives include a comparison of direct costs between different mechanisms of injury, survivors and fatalities; use and non-use of safety devices; and by age groups, gender or occupation. This study will contribute to the knowledge base for healthcare prioritization, strategic planning and future occupational safety efforts in Qatar and in other similar settings.

## 2. Materials and Methods

A retrospective analysis of data obtained from the trauma registry of the HTC, the national Level 1 trauma referral center of Qatar, was conducted. The study duration was from 1 January 2011 to 31 December 2017, and it included all working-age (>17 years) patients with moderate-to-severe WRIs in the registry. Patients that died before reaching the hospital were excluded. All patient data were anonymized prior to encoding and analysis.

The HTC is the only Level 1 trauma center serving the entire country, receiving more than 98% of the county’s trauma patients. The trauma registry at the study institution is the Qatar National Trauma Registry, which is compliant with both the National Trauma Data Bank (NTDB) and Trauma Quality Improvement Program (TQIP) of the American College of Surgeons Committee on Trauma (ACS COT); it collects data on all trauma activations and all trauma patients without trauma activation who are admitted to the trauma service with International Classification of Diseases, Ninth Revision (ICD-9) codes between 800 and 959.9. Moderate-to-severe WRIs were defined as injuries suffered during working hours or while traveling to or from work and severe enough to need admission to the hospital; work-related burns, drowning, poisoning or heat-related illnesses were excluded, as they are not treated at the HTC.

Variables collected and analyzed included patient demographics, type and mechanism of injury and road-user type. Severely injured patients who died at the scene before arriving to the hospital and those with mild injuries who were seen, treated and discharged from the Hamad Medical Corporation (HMC) Emergency Department, Qatar Red Crescent Clinics (QRC) or at Primary Health Care Centers (PHCCs) were excluded from the study.

The cost of WRIs in this study was calculated by using a micro-costing methodology [18], including multiplying the number of procedures, radiology tests and hospital stays (HLOS) with the respective unit costs. The overall direct health-service cost was estimated as the sum of the HLOS cost, procedure cost and radiological cost. All costs were represented in US dollars (USD). The Qatari riyal (QAR) was pegged to the USD at the exchange rate of 3.64 QAR per one USD since 2001, following the law which states that the currency will be maintained within a band between 3.6385 and 3.6415 QAR per USD [19]. The institutional medical cost was obtained from the “Estimated Cost of Service—Summary”, Cost Accounting Section, Finance Department, Hamad Medical Corporation, Doha, Qatar. This document lists hospital costs for various diagnostic tests, operative procedures and hospitalization for patients based on their admission diagnosis. The national data on labor-force population, health spending and budgets were accessed from official government institutions.

This study obtained ethical approval from Research Ethics Committee, at Medical Research Center, Hamad Medical Corporation (HMC), Doha, Qatar (“Unified Registry for Workplace Injury Prevention in Qatar” (WURQ), Grant (NPRP 7-1120-3-288)).

### Statistical Analysis

Descriptive and inferential statistics were applied for data analysis. Number of patients in each category was presented, along with valid percentages. Cost estimates were presented as point mean with standard deviation, while length of stay was presented as median with interquartile range (IQR). One-way ANOVA was used to compare the cost associated with the procedure, HLOS, radiological or overall cost by age-groups, mechanism of injury and occupation. Student’s *t*-test was used to compare the mean costs by gender, helmet use, seatbelt use and survival. Categorical variables were compared by using chi-square test. Non-parametric testing was used to compare the medians across the groups, such as hospital LOS. Bonferroni correction was used, and *p*-value was taken as significant when *p* < 0.05 (two-tailed). Multivariable linear regression analysis was performed to determine the association between different costs (overall cost, HLOS cost, procedure cost and cost for radiology). Statistical Package for the Social Sciences (SPSS) for Windows V.21.0 (SPSS, Chicago, IL, USA) was used for the data analysis.

## 3. Results

A total of 3757 WRI patients were admitted to the national trauma center during the 7-year study period; they accounted for 31.3 % of all trauma admissions (see Appendix A: Patient data flowchart). The mean age of patients was 34.6 ± 10.3. Most (38.0%) of the patients were 18–29 years of age, followed by 30–39 years (33.2%). Males made up almost all the WRI victims (98.3%), and general laborers were the most common occupation injured (44.3%). The most common mechanism of WRI was falls from height (47.7%), followed by motor vehicle crashes (MVCs) (19.0%) and falling of heavy objects or hit by objects (16.9%). Seatbelts were used by only 42.9% of the passengers and drivers injured in MVCs. Helmets were used by 68% of patients with motorcycle- or bicycle-related injuries (Table 1).

The overall cost, from 2011 and 2017, incurred for the patients with WRIs was estimated at 124,671,431 USD, i.e.,17.8 million USD per year (Table 1). There were no significant differences in median overall cost by age groups, gender or occupation. The median overall cost was significantly higher for pedestrian injuries (25,308 USD) and MVCs (22,207 USD) when compared to other mechanisms of injury. The median overall cost incurred for the patients involved in MVCs who were not using seatbelts was significantly higher than for those who used seatbelts (28,873 vs. 15,645 USD, *p* = 0.001).

The injury severity score (ISS) and hospital LOS (HLOS) were comparable between different age groups and genders. However, pedestrian injuries were more severe and associated with a longer HLOS. Moreover, patients who did not use seatbelts in MVC-related injuries had a significantly higher ISS and longer HLOS than those who used seatbelts (ISS, 15.3 vs. 11; HLOS, 9 vs. 6 days; *p* = 0.001). Although general laborers suffered more severe injuries than other occupational groups, the HLOS duration was comparable (Table 1).

Nearly 5% of the total cost was spent on patients with fatal work-related injuries (Table 2). Cost associated with the HLOS (60%) was the major contributor of total cost in the patients discharged home whereas procedure cost was the main contributor in total cost of treatment for patients who died in the hospital (61%). However, the median total cost differences between non-fatal and fatal injuries were statistically insignificant (*p* = 0.08). The ISS for mortalities was significantly higher (31 vs. 12, *p* = 0.001). The HLOS for survivors was significantly longer (7 vs. 2, *p* = 0.001).

Overall, the HLOS cost accounted for 58% and procedure costs 39% of the total. There was a strong positive correlation between the overall cost and hospital stay cost (r^2^ = 0.949, *p* = 0.00001) and between the overall cost and procedure cost (r^2^ = 0.852, *p* = 0.00001). Other variables showed a weak correlation (r^2^ < 0.40).

In a comparison of key characteristics for WRIs broken down by costs by quartile, no significant differences were noted by occupation (Table 3). Victims of pedestrian and motor vehicle crashes and those under 30 years of age made up a significantly higher proportion of the patients who generated costs in the highest quartile.

Table 4 demonstrated that HLOS accounted for 58% of the total cost and approximately 40% was expended for procedures. From 2011 to 2017, the overall procedure and radiology costs increased by 62.4% and 111% respectively. The r^2^ values for trendline for procedure and radiology costs were 0.77 and 0.82, respectively. On the other hand, the HLOS cost decreased by 23% in the study duration, and the r^2^ value for the trendline was 0.03. The total cost increased by 5% over a 7-year period (r^2^ = 0.31).

## 4. Discussion

The present study used the “best-evidence” approach to arrive at an estimate for the direct healthcare costs of inpatient care for WRIs in Qatar. It demonstrates that a focused work-related injury registry is required for more precise healthcare cost estimations, as much of the required information was not available. The findings highlight two major occupational health issues at hand: (1) young migrant workers in high-risk occupations are the main victims and (2) road traffic injuries, as well as falls, are the biggest contributors of WRI burden. These findings are consistent with previous reports [12], and both are wholly amenable to proven preventive measures, such as mandatory PPE and seatbelt use.

The study demonstrated that WRIs resulted in significant direct healthcare costs in Qatar; from 2011 to 2017, nearly 125 million USD was spent as direct healthcare costs to treat WRIs. This is almost double the combined annual direct medical costs for breast and colon cancer, as estimated for Qatar in 2019 [20]. Most of this expenditure was for the HLOS (58%), followed by the procedures performed during their treatment (39%).

There were no significant differences in overall cost for WRIs in terms of age groups, genders, occupation or patient outcomes (dead vs. alive). However, pedestrian injuries and MVCs at work had significantly higher costs than the other, more common mechanisms of injury, including falls and falling objects. Seatbelt use was found to reduce the overall cost of MVC injuries, as well as the severity of injuries and mortality rates; however, helmet use was not associated with a similar reduction in motorcycle and bicycle injuries.

The proportionate mortality, 12.5% of all deaths in 2010, from road-traffic injuries (RTIs), including pedestrian, motorcycle and bicycle injuries, in Qatar led to recommendations to make road safety a national priority [21]. In 2013, the World Health Organization Eastern Mediterranean Regional Office (WHO-EMRO) identified the lack of a comprehensive mandatory seatbelt law as one of the key deficiencies in the road-safety profile of Qatar [22]. In 2016, El-Menyar et al. clearly reported that the nonuse of seatbelts is associated with worse outcomes during MVCs in Qatar and recommended a comprehensive mandatory seatbelt law [23]. In a 2018 WHO road safety-status report, this same deficiency was still noted [24]. Over 86% of the population of Qatar is made up of expatriates, i.e., migrant workers, and their urgent healthcare needs are provided for free at government hospitals [25]. It is hoped that the findings from this study will provide the evidence needed to support the enactment of a comprehensive seatbelt law for all vehicle occupants, as it will provide local evidence of its cost-effectiveness, more so in a country with a majority of expatriate workers whose healthcare needs are fully paid for by the government.

The data on seatbelt utilization were only analyzed for the patients who were involved in an MVC as a vehicle occupant. These data were taken from the report by ambulance personnel and further confirmed by the trauma registry staff who interviewed the patients themselves, when and if the patient was able. Patients who were unable to respond or had “unknown” seatbelt usage were included in the “no seatbelt” group. This may have imparted a selection bias to classify patients with more severe injuries in the “no seatbelt” population, but the basis for this has already been proven in Qatar [23].

Patients with significantly more severe injuries obviously stayed longer in the hospital, resulting in significantly higher costs. In other words, more consistent and effective implementation and enforcement of road-safety laws could have saved more than $30 million during the study period, in addition to saving lives and preventing life-long disabilities.

On the other hand, patients who died at the hospital had more severe injuries with a significantly higher ISS and a shorter HLOS without a significant cost difference when compared to survivors. However, this statistically “insignificant” cost difference, of over 7000 USD more in the median costs for fatal WRIs, must be studied in greater detail. Most of the direct hospital costs of caring for fatal WRIs in Qatar are generated in the context of the early resuscitation and care of these severely injured patients within the first few hours of their arrival at the trauma center. In Qatar, costs for all forms of acute and urgent care are provided free of charge and borne entirely by the government; even the most severely injured are fully treated [25]. The aggressive clinical protocols for resuscitation followed at the Hamad Trauma Center [26] are demonstrated by the significantly greater proportion of total costs from procedures for non-survivors (*p* = 0.001). Regardless of nationality, all victims of injury are treated aggressively and equally, using best practice and evidence-based protocols [26].

This study also shows that general laborers suffered more severe injuries than other occupational groups; this finding has multiple implications for investments in workplace safety, better training of general laborers about injury prevention, provision of personal protective equipment where required and regular inspection of safety standards at the workplace. These measures would not only help by decreasing the number and severity of WRIs, but also direct and indirect costs associated with treatment, rehabilitation and repatriation.

The main strength of our study was that the data were obtained from the trauma registry of the national trauma center which prospectively records and maintains information about every trauma patient treated. Our institution treats more than 98% of all moderate to severely injured trauma patients in Qatar, and therefore the data obtained were nationally representative. The trauma center is compliant with globally accepted standards for the triage, admission, data encoding and care of trauma patients [26]. This study however does not represent the less severely or mildly injured victims of WRIs, who may still generate considerable healthcare costs from any long-term sequela of their injuries.

Cost analysis studies in WRIs are needed for the justification and planning of budgets, establishment of preventive and interventional programs and prioritization of research funding by healthcare policymakers. The overall healthcare costs in this study were estimated based on the incidence of the WRIs, analyzing data from the national trauma center that provides care for all cases with moderate and severe WRIs in the country, as this is one of the recommended approaches in health economics [18,19,20]. Medical costs are further classified into direct (types of payments and expenses) and indirect (resource utilization) costs. The direct cost involves costs incurred for in-hospital and outpatient services, medical supplies, laboratory investigations, medication, rehabilitation services at care centers and/or home, and caregiver costs [27,28,29].

Table 5 describes five previous studies which estimated the healthcare costs for WRIs to some extent [17,27,30,31,32]. Leigh et al. used the incidence method and was based on the estimates of average costs of WRIs and ill health from the literature [31]. Biddle reported the healthcare costs associated with fatal WRIs based on the Detailed Claims Information database [32]. Safe Work Australia adopted the incidence approach to report the healthcare costs for WRIs and ill health in workers and families in Australia. This included medical costs and the costs of care, aids and modifications that were uncompensated by the insurance company or the government. Costs were subcategorized by the severity level of injuries [27]. The Health and Safety Executive (HSE) of the United Kingdom also estimated the healthcare costs for WRIs and illnesses (excluding occupational cancer and other long-latency diseases) for workers and families and the government [33]. The HSE used the Labour Force Survey (LFS) data, published data and expert opinion for the estimation of healthcare cost [33]. Unfortunately, these estimates do not focus solely on severe WRIs and are not easily amenable to comparison to the estimates from this study.

Costs associated with falls are of main concern, as these injuries account for nearly 44% of the overall cost in our study, i.e., 7.8 million USD per year. Tuma et al. [17] concluded that the total cost associated with the work-related falls was 4.4 million USD per year in Qatar, and the mean cost was 15,735 USD per patient per year in 2007/2008. The mean cost associated with fall injuries in our study was 31,271 USD. In-hospital mortality was almost twice among fall-injury victims in the previous study (9.7% vs. 5.2%), and the study period was before the implementation of standardized pre-hospital care, trauma resuscitation and treatment protocols; this could explain the mortality and cost difference when compared to the present study.

Table 4 demonstrates a significant increase in procedure costs over the years (R^2^ = 0.92). The reduction in the mortality rate for victims of falls from height can be attributed to the many quality improvement efforts implemented in the Qatar trauma system; these have been described elsewhere [26]. The fact that pre-hospital deaths were excluded from this analysis can partially explain the 15,536 USD increase in the mean cost per case of WRI from a fall, compared to the previous report, where 58% of all deaths occurred in the pre-hospital setting [17].

The retrospective nature of the data-collection process is the major limiting factor of the current study. Moreover, patients who were treated and discharged by the emergency department were not recorded by the trauma registry, and, therefore, this study only describes costs incurred by moderate and severe WRIs. This may underestimate the true total cost of WRIs in Qatar. This study did not account for out-of-pocket costs, direct non-medical costs, payments incurred by the patients or other indirect costs. All of these clearly contribute to the underestimation of the total costs, but this study focused on the direct medical costs only. Pharmacy-related and rehabilitation costs and non-medical direct expenses were not accounted for the analysis, due to lack of sufficient data. We could not estimate the indefinite costs involving depression, distress, pain, suffering and stress caused by injury. Moreover, the indirect costs of WRIs from the lost productivity on the part of the patient and costs associated with work loss hours, residual disability or even caregiver time were not taken into consideration. This cost analysis study has other limitations, such as the lack of information about the cost of outpatient care and use of resources for chronic conditions, such as hospital or home-based rehabilitation after injury. We attempted to remove uncertainty and provide locally relevant cost data by using the most accurate and standardized direct cost estimates available.

The injury prevention perspective of this study focuses on demonstrating the cost-effectiveness of the use of proven protective devices, such as seatbelts, in work-related MVCs. Increasing seatbelt use by workers in Qatar will result in a significant reduction in the MVC victim’s severity of injury, death as well as healthcare costs. From this analysis, for every 22 workers who use a seatbelt on the job, or in work-provided transportation, one MVC death is prevented.

Other mechanism of WRIs did not lend themselves to such a direct demonstration of cost-effectiveness. WRIs affecting bicycle and motorcycle users who used helmets were not significantly different from those who did not.

The database used in this study did not provide consistent data on the use or non-use of proven safety devices, such as harnesses or hardhats, for victims of falls or falling object injuries. As such, no conclusions on the cost-effectiveness of either safety device could be made.

## 5. Conclusions

The direct medical costs associated with work-related injuries in Qatar amounted to over 125 million USD over this study period. Procedures and hospital length of stay are the major drivers of overall cost. Nearly 44% of the cost was incurred by patients injured by falls. However, the mean overall cost associated with the treatment of WRIs incurred as pedestrians, drivers or passengers was significantly higher, and road safety should also be an area of focus for occupational safety efforts. Using seatbelts significantly reduced the severity of injury, deaths and costs for WRIs sustained in MVCs. These findings suggest that targeting these mechanisms of injury through proven programs and focused research should be priorities for worker safety in Qatar.

## Figures and Tables

**Table 1:** Median costs incurred, injury severity and hospital length of stay by different patient groups with work-related injuries, Hamad Trauma Center, Qatar (2011–2017).

Variables	*n* (%)	Overall Cost (USD)	Median Cost (IQR) (USD)	*p*-Value	ISS (Mean, SD)	*p*-Value	Median HLOS (IQR)	*p*-Value
All WRIs	3757 (100%)	124,671,431	19,071 (8688–38,005)	-	12.8 (9.8)	-	6 (3–11)	-
Age group, *n* (%)								
18–29	1406 (38.0)	49,189,825	19,999 (9735–40,572)	0.06	12.7 (9.6)	0.57	7 (4–15)	0.87
30–39	1228 (33.2)	3,857,0346	19,384 (9582–36,718)		12.5 (9.2)		7 (4–14)	
40–49	705 (19.0)	23,390,186	17,689 (6829–36,678)		12.4 (9.3)		6 (3–14)	
50–59	296 (7.9)	9,764,772	17,462 (7862–34,230)		13.3 (10.2)		6 (3–11.5)	
≥60 years	68 (1.8)	2,904,468	19,989 (7558–50,182)		12.9 (7.6)		8.5 (4.5–19.5)	
Sex, *n* (%)								
Males	3695 (98.3)	122,586,916	18,960 (8672–37,929)	0.16	12.8 (9.8)	0.78	7 (4–14)	0.91
Females	62 (1.7)	2,084,515	24,772 (10,399–40,091)		12.4 (11.9)		10.5(4–23)	
Mechanism of injury, *n* (%)								
Fall	1792 (47.7)	54,685,556	18,732 (7768–36,219)	0.001	13.3 (9.6)	0.001	7 (4–14)	0.001
Motor vehicle crash	714(19.0)	30,309,614	22,207 (9753–50,030)		13.5 (9.9)		7 (4–16)	
Falling or hit by heavy object	636 (16.9)	19,941,604	19,672 (9436–37,208)		12.6 (9.4)		7 (4–13)	
Machine-related	159 (4.2)	4,425,805	17,290 (11,748–27,689)		7.9 (5.9)		4 (3–9)	
Pedestrian	157 (4.2)	7,091,723	25,308 (11,821–56,945)		14.6 (11.5)		7 (4–19)	
Others	299 (8.0)	8,217,129	16,067 (7893–30,757)		10.5 (10.9)		5 (3–9)	
^1^ Seatbelt use, *n* (%)								
Yes	306 (42.9)	9,387,647	15,645 (5981–29,886)	0.001	11.0 (7.6)	0.001	6 (3–11)	0.001
No	408 (57.1)	20,921,967	28,873 (12,689–64,139)		15.3 (10.9)		9 (4–24)	
^2^ Helmet use, *n* (%)								
Yes	34 (68.0)	1,548,275	17,519 (11,322–32,586)	0.13	11.1 (9.8)	0.43	5.5 (3–6.5)	0.60
No	16 (32.0)	289,876	19,071 (8765–37,743)		8.9 (7.5)		4.5 (3–10)	
Occupation, *n* (%)								
General Laborer	1662 (44.3)	55,570,151	19,879 (9136–39,527)	0.18	13.3 (9.8)	0.03	7 (4–14)	0.21
Transportation and material moving	693 (18.5)	2,471,6743	18,375 (8155–41,717)		12.7 (9.3)		6 (3–14)	
Installation, maintenance, repair	313 (8.3)	10,723,040	19,276 (10,220–37,616)		11.8 (8.4)		7 (4–14)	
Carpenter	236 (6.3)	6,909,124	19,077 (9433–33,346)		11.8 (8.7)		7 (4–14)	
^3^ Others	849 (22.6)	26,703,502	17,311 (7699–34,006)		12.6 (10.9)		6 (3–12)	

USD: US dollars; SD: Standard deviation; IQR: Interquartile range; ISS: Injury Severity Score; HLOS: Hospital length of stay; ^1^ Seat-belt use denominators = motor vehicle crashes; ^2^ Helmet use denominators: bike and motorcycle-related; ^3^ Other occupations include 326 (8.7%) undocumented, 64 (1.7%) housekeepers, 61 (1.6%) building and grounds cleaning and maintenance, 57 (1.5%) farming, fishing and forestry.

**Table 2:** Comparison of median costs incurred, injury severity and hospital length of stay by in-hospital outcomes in patients with WRIs (*n* = 3757).

In-Hospital Outcome	*n* (%)	Cost Details (USD, %)	Median Cost (IQR) (USD)	*p*-Value	ISS (Mean, SD)	*p*-Value	Median HLOS (IQR)	*p*-Value
Alive	3577 (95.2)	Total cost: 117,637,434 (94.4) Hospital stay cost: 70,111,917 (59.6) Procedure cost: 44,918,654 (38.2) Radiology cost: 2,606,863 (2.2)	18,694 (8782–37,245)	0.08	12.0 (8.5)	0.001	7 (4–14)	0.001
Dead	180 (4.8)	Total cost: 7,033,997 (5.6) Procedure cost: 4,301,586 (61.2) Hospital stay cost: 2,622,539 (37.3) Radiology cost: 109,972 (1.6)	26,159 (3407–51,330)		31.0 (16.7)		2 (1–7)	

USD, US dollars; SD, standard deviation; IQR, interquartile range; ISS, injury severity score; HLOS, hospital length of stay.

**Table 3:** Comparison of patient characteristics by costs associated with work-related injuries by quartile, Hamad Trauma Center, Qatar (2011–2017) (*n* = 3766).

Variables	Q1 (4447 QR) (*n* = 939)	Q2 (13,589 QR) (*n* = 939)	Q3 (25,801) (*n* = 939)	Q4 (64,492) (*n* = 939)	*p*-Value
Age group, *n* (%)					
18–29	317 (33.8%)	350 (37.3%)	359 (38.2%)	381 (40.6%)	0.02
30–39	287 (30.6%)	319 (34.0%)	327 (34.8%)	295 (31.4%)	
40–49	203 (21.6%)	173 (18.4%)	160 (17.0%)	168 (17.9%)	
50–59	81 (8.6%)	80 (8.5%)	70 (7.5%)	65 (6.9%)	
≥60 years	20 (2.1%)	12 (1.3%)	12 (1.3%)	24 (2.6%)	
Undocumented	31 (3.3%)	5 (0.5%)	11 (1.2%)	6 (0.6%)	
Mechanism of injury, *n* (%)					
Fall	485 (51.7%)	425 (45.3%)	468 (49.8%)	413 (44.0%)	0.001
Pedestrian	29 (3.1%)	36 (3.8%)	32 (3.4%)	60 (6.4%)	
Motor vehicle crash	166 (17.7%)	159 (16.9%)	161 (17.1%)	228 (24.3%)	
Falling or hit by heavy object	145 (15.4%)	166 (17.7%)	172 (18.3%)	153 (16.3%)	
Machine-related	30 (3.2%)	60 (6.4%)	39 (4.2%)	30 (3.2%)	
Others	84 (8.9%)	93 (9.9%)	67 (7.1%)	55 (5.9%)	
Occupation, *n* (%)					
General Laborer	402 (42.8%)	395 (41.3%)	428 (45.6%)	436 (46.4%)	0.06
Transportation and material moving	180 (19.2%)	174 (18.5%)	152 (16.2%)	187 (19.9%)	
Carpenter	53 (5.6%)	65 (6.8%)	69 (7.3%)	49 (5.2%)	
Installation, maintenance, repair	67 (7.1%)	89 (9.5%)	82 (8.7%)	75 (8.0%)	
Others	237 (25.2%)	224 (23.9%)	208 (22.2%)	192 (20.4%)	
Outcome, *n* (%)					
Alive	884 (94.1%)	927 (98.7%)	895 (95.3%)	870 (92.7%)	0.001
Mortality	55 (5.9%)	12 (1.3%)	44 (4.7%)	69 (7.3%)	

**Table 4:** Costs associated with work-related injuries by year, Hamad Trauma Center, Qatar (2011–2017) (*n* = 3766).

Year	Patients (*n*, %)	Procedure Cost (USD, %)	Radiology Cost (USD, %)	Hospital Stay Cost (USD, %)	Total Cost (USD, %)
2011	524 (13.9)	4,849,942 (9.9)	227,201 (8.4)	10,534,617 (14.5)	15,611,760 (12.5)
2012	545 (14.5)	6,076,536 (12.3)	267,483 (9.8)	9,828,495 (13.5)	16,172,514 (13.0)
2013	526 (14.0)	6,106,843 (12.4)	276,810 (10.2)	10,828,722 (14.9)	17,212,375 (13.8)
2014	498 (13.2)	6,841,288 (13.9)	371,508 (13.7)	10,292,561 (14.2)	17,505,357 (14.0)
2015	587 (15.6)	8,930,149 (18.1)	520,178 (19.1)	11,313,810 (15.6)	20,764,137 (16.7)
2016	582 (15.5)	8,541,140 (17.4)	574,758 (21.2)	11,850,231 (16.3)	20,966,129 (16.8)
2017	504 (13.3)	7,874,242 (16.0)	478,897 (17.6)	8,086,020 (11.1)	16,439,159 (13.2)
Total	3766 (100.0)	49,220,140 (39.5)	2,716,835 (2.2)	72,734,456 (58.3)	124,671,431 (100.0)

**Table 5:** Description of studies reporting costs associated with work-related injuries (2011–2019).

Study Name	Cases	Perspective	Country	Level	Accidents or Ill Health	Objectives	Policy Purposes	Key Findings
Leigh et al. (2001) [31]	Fatal and non- fatal injuries	Government, Workers and families, Society	California, USA	Macro	Accidents and ill health	Estimate direct and indirect costs associated with occupational injuries and illnesses in California in 1992	Calculate the contribution to total healthcare and determine how much of these costs are covered by compensation costs	Approximately 660 job-related deaths from injury, 1.645 million nonfatal injuries, 7079 deaths from diseases and 0.133 million illnesses are estimated to occur annually in the civilian California workforce. The direct ($7.04 billion, 34%) and indirect ($13.62 billion, 66%) costs were estimated to be $20.7 billion. Injuries cost $17.8 billion (86%) and illnesses $2.9 billion (14%).
Biddle (2004) [32]	Fatal injuries	Society	USA	Macro	Accidents (fatal injuries)	Developing a computerized costing model for calculating cost consequences of occupational fatal injuries	Provide policymakers with a tool to use in cost–benefit analysis of prevention strategies	1980–1997: Number of Fatalities = 103,845 Total Cost = $83,223,558,201 Mean Cost = $801,421 Median Cost = $816,811
Tuma (2013) [17]	Fatal and non- fatal injuries	Workers	Qatar	Macro	workplace-related fall from height	To find out the actual medical costs of occupational-related falls in Qatar	To provide a reference for establishing fall prevention guidelines and recommendations	The total cost in US dollars during the 1-year period was $4421,507, with a mean cost of $15,735 per patient; as a reference to this number, each day in the ICU costs about $1500.
Safe Work Australia (2018) [27]	Fatal and non- fatal injuries	Government, Workers and families	Australia	Macro	Accidents and ill health	To update the estimated cost of work-related injury and illness	To estimate the burden to economic agents as a percentage of the total costs	The cost of work-related injury and disease to the Australian economy is $61.8 billion for, representing 4.1% of GDP.
Health and Safety Executive (2019) [33]	Fatal and non- fatal injuries	Government, Workers and families, Society	United Kingdom	Macro	Accidents and illnesses (excluding occupational cancer and other long-latency diseases)	Perform an update of the Health and Safety Executive (HSE) cost model and use it to generate cost estimates for years up to 2017/18	Provide an estimate of the total costs for society, employer and employees of occupational ill health and injuries	The total cost of workplace self-reported injuries and ill health in 2017/18 was £15.0 billion. Ill health causes the biggest proportion of total costs at around 65% (£9.8 billion), with injury resulting in around 35% of total costs (£5.2 billion)
Current Study	Fatal and non- fatal injuries	Workers	Qatar	Macro	Workplace-related injury	To find out the work-related injury cost.	To provide a reference for establishing work-related injury prevention guidelines and recommendations	The overall cost for WRI was 124,671,431 USD. Mean overall cost was 33,134 USD (95% CI 32,610 to 35,686). Mean radiology cost was 719 USD, (95% CI 707 to 739). Mean procedure cost was 13,073 USD (95% CI 12,496 to 13,699). Mean hospital stay cost was 19,342 USD (95% CI 18,300 to 20,300).

**Figure A1:**
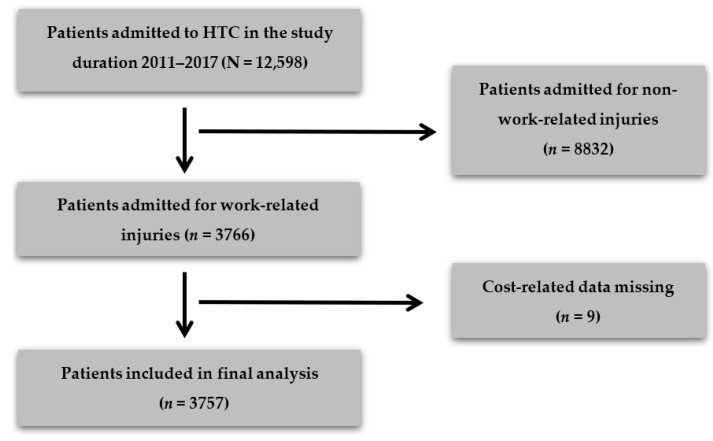
Patient data flowchart, Trauma Registry, Hamad Trauma Center, Doha, Qatar (2011–2017).

## Data Availability

Not applicable.
